# Removal of Hydrogen Sulfide in Septic Tanks for Treating Black Water via an Immobilized Media of Sulfur-Oxidizing Bacteria

**DOI:** 10.3390/ijerph17030684

**Published:** 2020-01-21

**Authors:** Jeong-Hee Kang, Hyeong-Gyu Namgung, Jeong-Il Cho, Sung Soo Yoo, Bong-Jae Lee, Hyon Wook Ji

**Affiliations:** 1 Department of Land, Water and Environment Research, Korea Institute of Civil Engineering and Building Technology, 283 Goyangdae-Ro, Ilsanseo-Gu, Goyang, Gyeonggi-Do 10233, Korea; kangjeonghee@kict.re.kr (J.-H.K.); jicho@kict.re.kr (J.-I.C.); yoosungsoo@kict.re.kr (S.S.Y.); bjdream@kict.re.kr (B.-J.L.); 2 New Tech. Research Department, Korea District Heating Corporation, 92, Gigok-Ro, Giheung-Gu, Gyeonggi-Do 17099, Korea; nghgna@kdhc.co.kr

**Keywords:** hydrogen sulfide, septic tank, odor management, black water, bioreactor

## Abstract

In South Korea, the installation of septic tanks for treating black water (STBW) is regulated even in sewage treatment areas to prevent the black water deposition in combined sewers. STBWs in which black water is anaerobically decomposed generate high concentrations of hydrogen sulfide (H_2_S). In this study, an immobilized media of sulfur-oxidizing bacteria (SOB) was used to remove the H_2_S. SOB media was prepared by using activated sludge collected from a wastewater treatment plant. Prior to field application, an appropriate cultivation period and aeration rate for SOB activation were estimated through a laboratory-scale test. The SOB was activated after a 23-day cultivation period and an aeration rate of 0.25 L-water/L-air/min. Moreover, the maximum H_2_S removal efficiency was observed at a cultivation period of 43 days and an aeration rate of 0.38 L-water/L-air/min. Then, the SOB media was installed on STBWs of various capacities. The H_2_S removal efficiency was compared between with and without SOB media. The maximum H_2_S elimination capacity with SOB media was 12.3 g/m^3^/h, which was approximately three times higher than without SOB media. Furthermore, the energy efficiency and oxidation rate were also three times higher with SOB, demonstrating the applicability of SOB for H_2_S removal in STBW.

## 1. Introduction

Septic tanks are on-site private sewage treatment facilities often employed in rural areas where it is difficult to send domestic wastewater to wastewater treatment plants (WWTP). Therefore, septic tanks are designed to separate solids and remove organic matter from domestic wastewater [1]. However, due to the poor conditions of the sewage system in South Korea, installing a septic tank for treating black water (STBW) is regulated in areas with combined sewers to avoid deposition of wastewater solids, odors, and hygienic problems caused by the direct discharge of black water into the sewage system.

STBWs are generally operated in anaerobic conditions because, in contrast with the aerobic method, the operational cost or post-installation management is not required except for cleaning once a year [2]. Black water remains in the STBWs for a long residence time of 2–3 days, during which various contaminants are decomposed by anaerobic bacteria [3]. In this process, the sulfur and nitrogen compounds in the water are decomposed together with organic matter, and various odors are generated in the STBWs [4]. Among the various odorous substances, hydrogen sulfide (H_2_S) is one of the major contributors because the generated concentrations are far higher than the minimum detectable concentration. For example, Zuo et al. [5] reported that H_2_S was generated at concentrations of up to 100–400 ppm_v_ in septic tanks. The odor of H_2_S due to its high concentrations in the STBWs spreads to sewers and reaches the surface through inlets, becoming a nuisance and a health hazard to urban residents. Therefore, STBW odors can be eliminated efficiently by removing H_2_S. In light of the above situation, the Korean Government mandated the installation of aeration systems to prevent anaerobic decomposition taking place in STBWs, and therefore, prevent the generation of odors. However, this method has a low H_2_S removal efficiency and cannot fundamentally solve odor problems because most of the H_2_S in the water evaporates into the atmosphere during aeration. Moreover, once the aeration pump operation is stopped, odors can easily reoccur because the STBW quickly becomes anaerobic again.

Various technologies to treat H_2_S have been developed and applied [6,7]. The physical treatment technologies, such as absorption and adsorption, require a large space and are cost-intensive. The chemical treatment methods have disadvantages such as difficulty of transport, requirement of chemicals, and high cost. Moreover, these methods are impractical to apply in a septic tank due to maintenance issues [8,9]. However, the application of biological treatment technologies is feasible because these require simple auxiliary installations and are easy to operate [10].

In a biological treatment process, the selection and cultivation of microorganisms that utilize the target substrate as a main metabolite is essential. To remove H_2_S, sulfur-oxidizing bacteria (SOB) is widely used. Because SOB use H_2_S as their primary substrate, they exhibit a high oxidation rate and high specific growth rate in the presence of this compound. Thus, these have been used in various biological treatment processes. There are many studies on removal and purification of H_2_S using SOB. Namgung and Song [9] developed a system to remove H_2_S in biogas by oxidizing it using *Thiobacillus*, resulting in a ≥98% H_2_S removal efficiency even at pH values below 1.5. Moreover, Yousef et al. [11] used SOB to treat industrial wastewater, and SOB has been used in many other instances and conditions to remove H_2_S [12]. Recently, a study on a SOB scrubber to remove high concentrations of H_2_S in sour gas even at an extreme condition of halo alkaline was reported [13].

However, studies involving the application of SOB to remove H_2_S in septic tanks are largely lacking. Neisi et al. [4] conducted experiments to remove H_2_S in septic tanks using a vermicomposting biofilter. They investigated the relation between gas retention time and removal efficiencies and long-term performance. However, H_2_S was treated using a biofilter in a separate space (not in the septic tank). As stated above, installation of new facility is impossible due to the confined space of septic tank and difficulty of maintenance. On the other hand, in the present study, the installation of SOB media in a septic tank was feasible because it is directly submerged in water, and among auxiliary installations, only a pump and a few pipelines are required.

SOB remove H_2_S by oxidizing it to form sulfate ions; therefore, we developed an immobilized SOB media. Operation parameters must be evaluated by applying SOB media to field septic tanks to obtain an effective H_2_S removal rate. The most important operation factors in biological treatment processes are aeration rate, quantity of nutrients and substrates, temperature, and pH. Among the parameters, aeration rate is one of the most crucial factors because oxygen is an important electron acceptor for the metabolism of SOB [4,14].

Therefore, SOB-immobilized media were developed and applied in a septic tank. To estimate an optimized aeration rate, various experiments were conducted at laboratory-scale and pilot-scale setups. SOB media was prepared by cultivating SOB in commercial Styrofoam. It was installed inside a STBW and oxygen was supplied. To determine the basic parameters for the application of SOB to STBW, the SOB activation period (i.e., the culture time required to observe SOB activity) was determined, and the H_2_S removal efficiency was verified via a laboratory test. Additionally, the SOB media was installed in real field STBWs to assess their H_2_S removal rate (odor elimination capacity); this served as an indicator of their operational efficiency.

## 2. Materials and Methods

### 2.1. Preparation of Sulfur-Oxidizing Bacteria (SOB) Media

**Figure 1:**
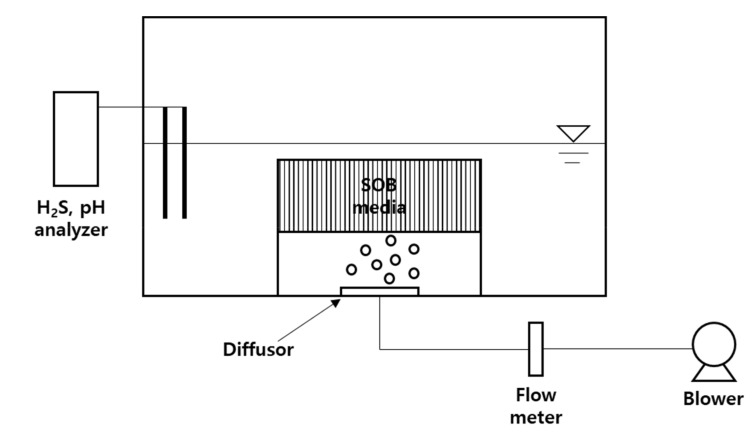
Schematic diagram of the experimental setup for sulfur-oxidizing bacteria (SOB) media in laboratory-scale tests.

Figure 1 illustrates the experimental setup for the preparation of SOB media. The SOB media was cultivated in a Plexiglas reactor with dimensions 380 × 580 × 690 mm. A commercial Styrofoam was embedded in the reactor as a substrate for SOB attachment. The Styrofoam, which has the porosity of 40 PPI, was cut into 1 cm^3^ cubes and inserted into the SOB culture to prepare the SOB media. A disk-type ceramic diffusor was installed at the bottom of the reactor to inject fine air bubbles.

Activated sludge and wastewater collected from a WWTP were used as seed for SOB culture. The SOB culture was prepared by mixing sludge of 10 L and wastewater of 40 L, after which the SOB was cultivated by injecting air into the SOB culture at 10 L/min. The H_2_S concentration in the wastewater (i.e., the main substrate for SOB) was approximately 0.5 mg/L, which was insufficient for SOB growth. Thus, the SOB culture was replaced with 10 L of NaHS solution containing 150 mg/L of H_2_S every day. Before injecting the solution, the pump operation was stopped, and 10 L of the supernatant was extracted. As a carbon source for SOB growth, 10 g of milk powder was added every day. The pH of the SOB culture was maintained neutral at approximately 7.0 throughout the cultivation period.

### 2.2. Laboratory-Scale Experiment

The experimental setup for H_2_S removal in the laboratory was similar to the setup for SOB medium preparation depicted in Figure 1. A box containing 1 L of SOB media was placed inside an 80 L Plexiglas reactor (400 × 400 × 500 mm) by fixing the bottom of the box to the reactor. Synthetic black water containing H_2_S of 10 mg/L was prepared by using NaSH and distilled water. The inside of the reactor was filled with 40 L of synthetic black water. Air was injected into the SOB media from the bottom using an impeller pump; the airflow was adjusted to correspond to the aeration rate of 0.13–0.38 L-air/min/L-water using a flow meter. Liquid H_2_S and pH analyzers were installed to monitor H_2_S concentration and pH changes.

### 2.3. Pilot-Scale Test

**Figure 2:**
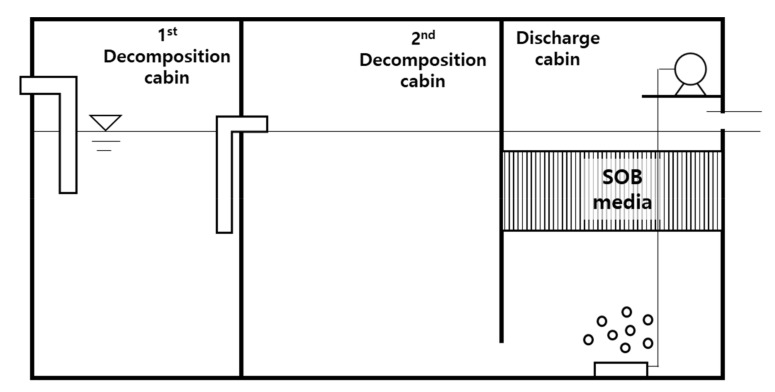
Sulfur-oxidizing bacteria (SOB) media applied in a septic tank for treating black water (STBW).

Figure 2 illustrates the SOB media installed in a typical STBW. The SOB media was installed in the last filtration tank in a 3-stage STBW. The media volume was 1 m^3^, and installation was carried out after a 40-day incubation period in the laboratory. Similar to the laboratory setup, the air was supplied by connecting a diffusor and a blower. The blower had a 2.2 kW capacity and was operated at its maximum output during the test period. The SOB media (with aeration) was placed in 20 STBWs in commercial buildings in Seongnam, Gyeonggi-do; all STBWs examined herein were varied in the range of 70–1000 m^3^ capacity.

### 2.4. Analytical Methods

The H_2_S concentration and pH in water were measured using an H_2_S analyzer (MS08, AMT Analysenmesstechnik GmbH, Rostock, Germany) equipped with an amperometric probe. After the completion of the test, the concentration was measured by directly immersing the probe in the liquid inside the reactor. The measured concentration range was 0–50 mg/L, with a detection limit of 0.5 mg/L. The gaseous H_2_S concentration was measured using an H_2_S analyzer (GHS-8AT, GASTEC Corporation, Kanagawa, Japan) equipped with a diffuse electrochemical sensor installed inside the STBW. The measured concentration range was 0–500 ppm_v_, with a detection limit of 1 ppm_v_.

The sulfate ions were measured using an ion chromatograph (792 Basic IC, Metrohm AG, St. Gallen, Switzerland) equipped with A-Supp 15 column. After the test was completed, some aqueous solution in the analyzer or STBW was collected for analysis.

## 3. Results and Discussion

### 3.1. SOB Activation Period

The inside of the STBW was anaerobic because it was isolated from the atmosphere, thus creating unfavorable conditions for SOB growth. Moreover, even though oxygen was supplied through a blower, it would have been unreasonable to expect an immediate increase in SOB activity. Therefore, the SOB activation time had to be estimated. For this, SOB media was prepared and cultivated for 5, 12, 23, 30, and 45 days, after which the H_2_S removal rate for each media was tested. The H_2_S removal rate was calculated using the following equation.

(1) $$\mathrm{Oxidation}\;\mathrm{rate}\;\left(\mathrm{mg}/h\right)=\frac{C_{0}-C_{t}}{t}$$

where C_0_ and C_t_ are the concentrations of aqueous H_2_S (mg/L) at the start and end of the experiment, respectively, and t is the experiment time (min). These rates were then compared with the H_2_S reduction rate when only aeration was performed without SOB media.

Prior to the evaluation of H_2_S removal rate, microbial identification analysis via 16S rRNA sequencing was performed on SOB media samples. The analysis results indicated that the top 20 dominant species identified accounted for 48%–71% of all microbes in samples. The most dominant microbe belonged to the family Rhodocyclaceae, which encompasses SOB species [15]; this indicates that H_2_S was likely oxidized by the SOB media.

**Figure 3:**
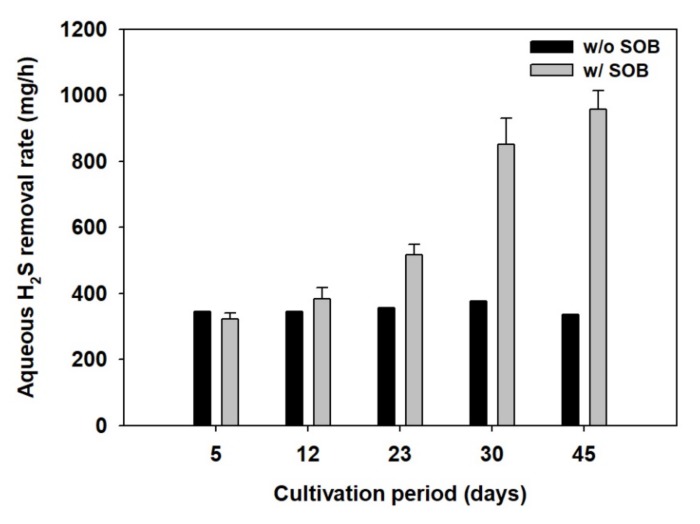
Variation of H_2_S removal rate depending on the cultivation periods. The error bars indicate the standard deviation of triplicate experiments.

The experiments showed that approximately 330 mg of H_2_S was removed per hour without SOB media (Figure 3). In the figure, w/o SOB and w/SOB represent without and with SOB, respectively. Moreover, applying SOB media cultivated for less than 12 days resulted in a similar result due to low SOB activity. However, when the SOB media cultivated for 23 days was applied, the H_2_S removal rate increased to approximately 580 mg/h. Furthermore, the H_2_S removal rate was 980 mg/h with a 45-day culture. Thus, it was concluded that SOB cultures exhibit activity after 23 days of cultivation, meaning that field applications would require no less than 23-day SOB cultures under aerobic conditions.

The H_2_S removal rate was 330 mg/h with aeration only, which was largely attributable to evaporation into the atmosphere via stripping. Table 1 summarizes the pH, sulfate ions, and H_2_S concentration of the aqueous solution after the end of the experiment. The total amount of sulfate ions remaining in the aqueous solution with aeration was approximately 32.1 mg, which was approximately 30% of the removed H_2_S amount. Although aeration only had a notable H_2_S reduction effect, most of the H_2_S was emitted into the atmosphere, which does not resolve the problem of H_2_S odors. In contrast, once SOB became activated after being cultivated for 23 days or more, more than 50% of the H_2_S was oxidized to sulfate ions. When the SOB were cultivated for 30 days or more, the oxidation rate was 64%. Thus, it can be concluded that H_2_S oxidation to sulfate ions after SOB media application would substantially reduce odors in STBWs. However, SOB media with less than 23 days of cultivation exhibited a low SOB activity and only a 30% H_2_S oxidation rate to sulfate ions, which was similar to that of aeration only. Therefore, SOB media should have sufficiently high activity to achieve effective odor removal from the STBWs.

The oxidation paths of aqueous H_2_S to solid sulfur or sulfate ions via SOB activity are detailed in Equations (2)–(4) [12]. However, protons are generated in the process of oxidation to sulfate ions, resulting in a pH decrease in the aqueous solution. This is demonstrated by the results in Table 1, which indicate that the pH was lowered at higher oxidation rates. When oxidizing H_2_S with 45-day SOB cultures, which exhibited the highest SOB activity, the pH decreased to 5.7. However, SOB exhibit their highest activity at a neutral pH; thus, constant low pH conditions during long-term operations could adversely affect the SOB activity, and countermeasures would be required to address this problem [16].

(2) $$H_{2}S+0.5O_{2}\to S^{0}+H_{2}O\;\left(\Delta G^{0}=-209.4\;\mathrm{kJ}/\mathrm{reaction}\right)$$

(3) $$S^{0}+1.5O_{2}+H_{2}O\to {\mathrm{SO}}_{4}^{2-}+2H^{+}\left(\Delta G^{0}=-587.1\;\mathrm{kJ}/\mathrm{reaction}\right)$$

(4) $$H_{2}S+2O_{2}\to {\mathrm{SO}}_{4}^{2-}+2H^{+}\;\left(\Delta G^{0}=-798.2\;\mathrm{kJ}/\mathrm{reaction}\right)$$

### 3.2. H_2_S Removal in a Lab-Scale Experiment

As mentioned above, SOB activity increased gradually after cultivation for 23 days, and after 45 days, the H_2_S removal rate was more than three times higher than that of aeration alone. However, if the oxygen supply stopped or was insufficient, the H_2_S removal efficiency decreased due to low SOB activity. Additionally, aeration rate is also one of the critical factors for SOB. Oxygen supply increased with increasing aeration rate because of the enhancement in mass transfer rate. Thus, different aeration rates (0.13, 0.25, 0.38 L-air/min/L-water) were assessed with 40-day SOB cultures to compare their effect on H_2_S removal rates.

**Figure 4:**
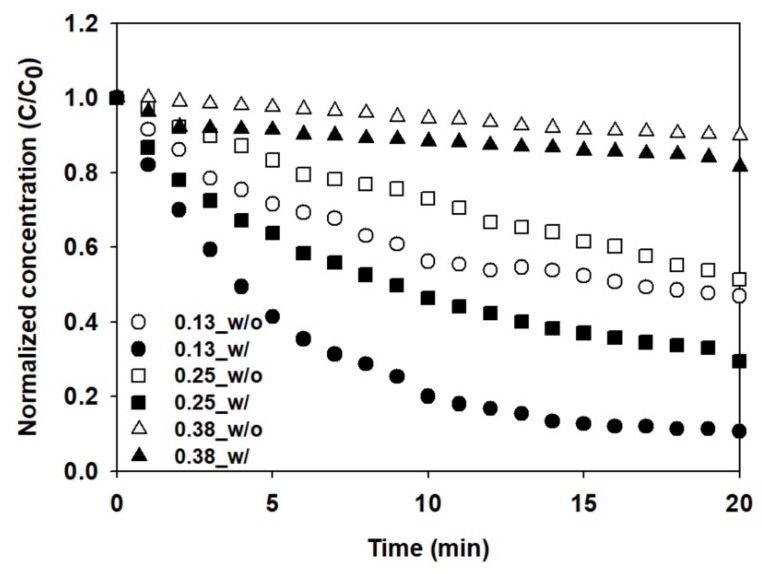
Changes in normalized H_2_S concentration with and without SOB media at different aeration rates.

Figure 4 shows the changes in H_2_S concentration in the water. The initial H_2_S concentration was approximately 10 mg/L. As the aeration rate increased, the concentration of aqueous H_2_S decreased faster, because the mass transfer rates of H_2_S from liquid to gaseous phase increased at higher aeration rates. Even with only aeration (without SOB), the removal rate of H_2_S was enhanced from 18% to 50% with increasing aeration rate. However, the aqueous H_2_S was mainly removed by stripping, and the H_2_S transfer rate from liquid to gas increased with under increased aeration rates.

The increased aeration rate also positively affected the H_2_S removal rate of SOB, indicating the mass transfer rates from gas to liquid phase. When the aeration rate was 0.13 L-air/min/L-water, the H_2_S removal rate was not substantially different from aeration only (without SOB). However, when the aeration rate increased to 0.25 L-air/min/L-water, the removal rate increased approximately 1.5-fold and almost doubled at 0.38 L-air/min/L-water. This is because the aqueous oxygen supply increased; proper dissolved oxygen (DO) levels were maintained by the higher mass transfer rate and, therefore, the SOB activity reached a maximum.

It is well known that SOB activated at DO above 0.5 mg/L. In addition, proper DO concentration could be achieved by means of operation at adequate aeration rate (Table 2). Cheng et al. [17] applied bioscrubber immobilized SOB to remove H_2_S in biogas. They reported that the concentration of DO was retained from 0.5 to 1.0 mg/L at an aeration rate of 0.28 L-air/min/L-water and obtained removal efficiency of 90%. Potivichayanon et al. [18] presented that a similar relation between aeration rate and DO concentration was observed. The aeration rate was 0.59 L-air/min/L-water and DO was maintained above 0.5 mg/L. Lohwacharin et al. [19] operated the airlift bioreactor with lower aeration rate of 0.07 L-air/min/L-water and DO was less than 0.2 mg/L. Rattanapan et al. [20] treated H_2_S in biogas using a packed bed biofilter at an aeration rate of 0.37 L-air/min/L-water and obtained removal efficiency of 100%. They concluded that oxygen was sufficiently supplied to the reactor because of an adequate aeration rate. Chaiprapat [16] also obtained a good removal rate of H_2_S by using the bioscrubber with an aeration rate of 0.29 L-air/min/L-water. Based on the reported literature, herein, the aeration rate of >0.25 L-air/min/L-water was used for adequate operation of the SOB media.

In the experiment without SOB, a linear reduction trend was observed for reduction of aqueous H_2_S concentration. This trend was due to the continuous H_2_S evaporation. This was because the evaporated H_2_S was continuously being discharged outside the reactor, and a gas–liquid concentration equilibrium could not be achieved. Accordingly, the driving force for mass transfer remained constant. Even with SOB media, a linear reduction trend was observed at 0.13 L-air/min/L-water because the oxidation performance was insufficient due to the low air supply. However, at an aeration rate above 0.25 L-air/min/L-water, the concentration decreased exponentially. This was presumably because the SOB activation and H_2_S available to the SOB decreased with the oxidation of H_2_S in water. This trend was more evident at 0.38 L-air/min/L-water, and almost no reduction of H_2_S concentration occurred after 15 min. Therefore, energy efficiency could be improved by adjusting the operation time of the blower based on the aqueous H_2_S concentration. This is an important advantage of the SOB media.

In addition, an increase in aeration rate improves mass transfer in both gas–liquid and liquid–gas directions, and this was likely the cause of the increased H_2_S removal rate [21]. Therefore, higher aeration rate led to higher SOB media performance among the experimental range herein.

### 3.3. SOB Media Application

Based on the lab-scale experiment, SOB media cultivated for 40 days was installed in the STBWs of two buildings with similar capacity (1.5 m^3^) and use (commercial complex). The liquid and gaseous H_2_S concentrations were observed for approximately 110 min, after which the aeration with and without SOB media were compared. The changes in the aqueous and gaseous H_2_S concentrations were monitored, and the operational index was calculated.

**Figure 5:**
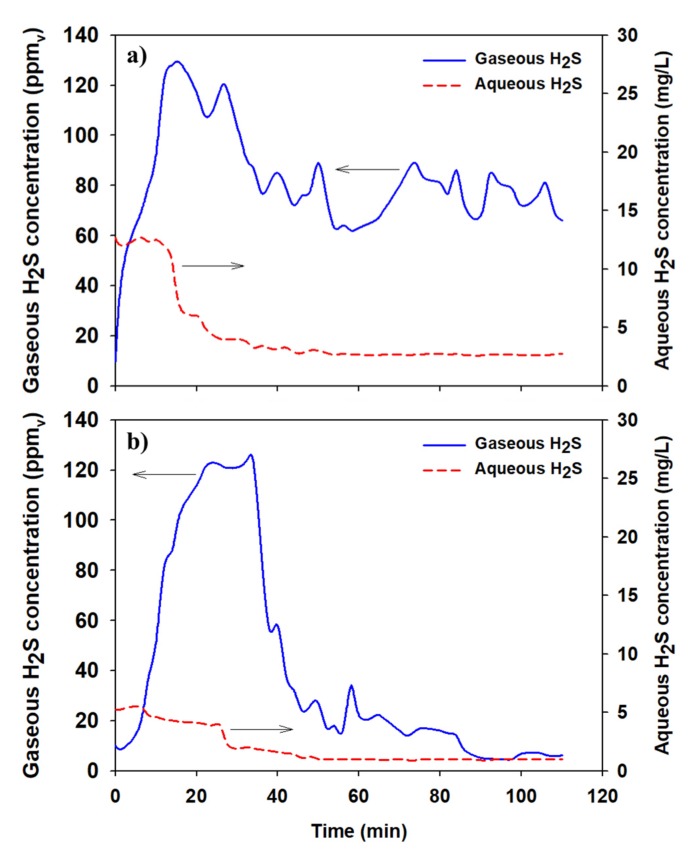
Concentration profiles of gaseous and aqueous H_2_S in (**a**) aeration without SOB and (**b**) aeration with SOB conditions.

Figure 5 shows a graph representing the concentration profiles of gaseous and aqueous H_2_S. When aeration was provided without SOB media, the initial H_2_S concentration of 10 mg/L in water remained constant for approximately 20 min before gradually decreasing. Although the evaporation of H_2_S occurred via stripping, high concentrations persisted. This was because the H_2_S in the sludge at the bottom of the STBW was disturbed by aeration. However, H_2_S in the water decreased, while H_2_S concentrations in the air continuously exhibited high concentrations. Additionally, H_2_S in the water was not oxidized due to the air injection and instead evaporated into the air. The H_2_S in the water remained at approximately 7 mg/L initially due to the disturbance of the bottom deposits, even with the addition of SOB. However, the concentration gradually declined to 1 mg/L at the end of the experiment, which was lower than that without SOB. Furthermore, the H_2_S concentration in the air also decreased sharply after 35 min and remained below 10 ppm_v_ thereafter due to the oxidation of aqueous H_2_S by SOB.

Considering the highest aqueous H_2_S concentration observed and the concentration at the end of the experiment, both experiments show similar removal efficiencies of approximately 95%. However, the H_2_S evaporated into the air was not oxidized into an odorless substance. Therefore, the net removal efficiency (RE_net_) must be calculated to account for the mass balance of aqueous and gaseous H_2_S concentrations. The equation for RE_net_ is detailed below:

(5) $${\mathrm{RE}}_{\mathrm{net}}\left(\%\right)=\frac{\left(C_{L,\mathrm{Max}}\times V_{L}+C_{G,\mathrm{Max}}\times V_{G})-(C_{L,\mathrm{end}}\times V_{L}+C_{G,\mathrm{end}}\times V_{G}\right)}{\left(C_{L,\mathrm{Max}}\times V_{L}+C_{G,\mathrm{Max}}\times V_{G}\right)}\times 100\left(\%\right)$$

where C_L,Max_ is the maximum concentration of aqueous H_2_S (mg/L), V_L_ is the aqueous volume (L), C_G,Max_ is the maximum concentration of gaseous H_2_S (mg/L), and V_G_ is the volume of the headspace (L). RE_net_ was 43.8% for aeration without SOB and 93.4% for aeration with SOB, respectively. Thus, SOB demonstrated a removal efficiency twice as high as that of aeration alone. In conclusion, SOB media has noticeable removal capacity for H_2_S in STBWs.

SOR (specific oxidation rate) is the amount of H_2_S that can be removed per power unit applied, i.e., the H_2_S reduction relative to the input power. It was calculated as follows:

(6) $$\mathrm{SOR}\left(g\cdot S/\mathrm{kWh}\right)=\frac{\left(C_{0}-C_{t}\right)\cdot V}{P\cdot t}$$

where C_0_ and C_t_ are the concentrations of aqueous H_2_S (mg∙S/L) at the start and end of the experiment, respectively, V is the volume of the aqueous solution (L), P is the power consumption of the blower (kW), and t is the experiment time (h). When only aeration was performed (without SOB), the SOR was 85.02 g∙S/kWh; in contrast, the SOR with SOB was approximately three times higher (242.26 g∙S/kWh). In conclusion, SOB technology has high potential applicability for STBW odor removal owing to its higher H_2_S removal and power efficiency, compared to that of aeration alone.

**Figure 6:**
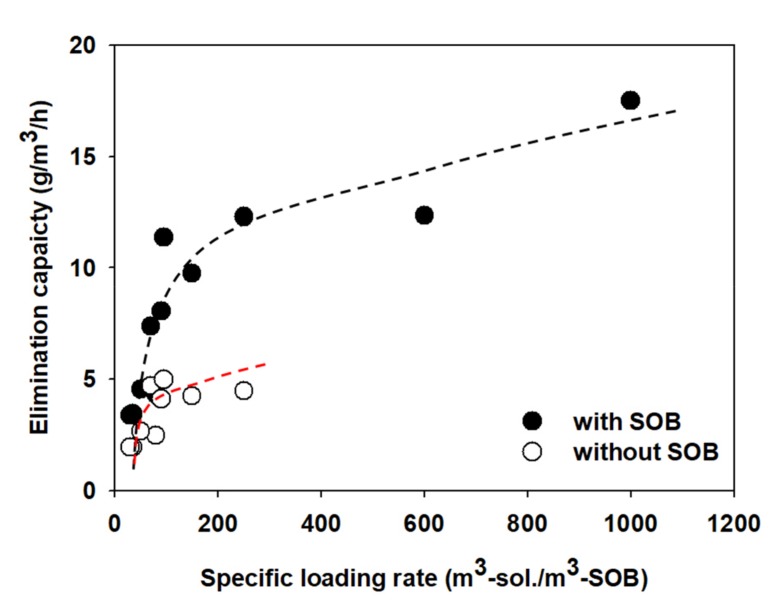
Short-term elimination capacity curves with and without SOB.

Proper aeration rate and power consumption were estimated to apply to the SOB media. Additionally, the volume of SOB media is also an important operational factor. The proper volume of the media can be determined by evaluating the maximum elimination capacity (EC) per SOB media unit volume. To determine the elimination capacity, the same 1 m^3^ media and 2.2 kW pumps were installed and operated at 20 STBWs with a capacity from 100 to 1000 m^3^. Then, the aqueous H_2_S concentration was measured 24 h after starting the operation. Figure 6 shows a graph representing the relationship between the specific loading rate (m^3^-solution/m^3^-SOB), which accounts for the STBW volume per 1 m^3^ of media, as well as the EC. The EC was calculated using the following equation:

(7) $$\mathrm{EC}\left(g/m^{3}/h\right)=\frac{\left(C_{0}-C_{t}\right)\cdot V_{L}}{V_{s}\cdot t}$$

where C_0_ and C_t_ are the concentrations of aqueous H_2_S (mg/L) at the start and end of the experiment, respectively, V_L_ is the liquid volume in the STBW (m^3^), vs. is the volume of the SOB media (m^3^), and t is the reaction time (h). When only aeration was performed, the maximum EC was approximately 4.5 g/m^3^/h; in contrast, the maximum EC in the STBW with SOB media nearly quadrupled to 17.5 g/m^3^/h. The EC in this study was very low compared to that reported in the literature. For example, Zytoon et al. [22] treated gas containing H_2_S at very high concentration of 10,000-25,000 ppm_v_ in an airlift bioreactor and reported that the maximum decomposition capacity was 111.3 g/m^3^/h. Moreover, Ramírez et al. [23] obtained a decomposition capacity of 20–140 g/m^3^/h depending on the gas retention time, with the inlet concentration of H_2_S gas being 372 ppm_v_. Aroca et al. [24] tested H_2_S removal rate using two biotrickling filters immobilized with *Thiobacillus thioparus* and *Acidithiobacillus*. The obtained EC of 14 g/m^3^/h with *Thiobacillus thioparus* at a H_2_S concentration of 400 ppm_v_, while the EC obtained with *Acidithiobacillus* was 300 g/m^3^/h at a 2000 ppm_v_. Chaiprpat et al. [16] conducted the study using the bioscrubber and reported EC of 220 g/m^3^/h at 8700 ppm_v_ H_2_S. In conclusion, EC is influenced by the quantity of substrate, i.e., H_2_S. Therefore, EC obtained in this study had smaller value than that reported in other studies due to lower concentrations of H_2_S in the septic tanks.

In addition, it would be difficult to directly compare the maximum decomposition capacity observed in this study with that of other bioreactors. Firstly, the SOB media applied to the STBW was operated in batch mode; when aeration is initiated, the conditions of the STBW become aerobic, and the generation of H_2_S is nearly suppressed. Therefore, once the H_2_S begins to be oxidized by SOB, only the residual H_2_S participates in the reaction. Furthermore, the reaction time considered for the calculation of EC in this study is likely higher than the actual time required to achieve the maximum EC. For instance, the concentration of aqueous H_2_S was likely to reach a steady state several hours earlier than end of the experiment. Accordingly, the actual reaction time would be less than 24 h, and thus the EC value must be underestimated. Therefore, the EC value calculated in this study must be applied only for calculating the volume of the media in the STBW and must not exceed 1000 m^3^-solution/m^3^-SOB, considering the capacity of the STBW.

## 4. Conclusions

An immobilized SOB media was applied to effectively remove H_2_S and ameliorate its associated odor from STBWs. To achieve this, operating factors were determined. The cultivation period was defined as the minimum period of 23 days required for SOB adaptation to the matrix, and 0.25 L-air/min/L-water should be applied to obtain the H_2_S removal rate. The cultivation period or aeration rate improved the H_2_S removal rate. In the pilot-scale tests, all three factors, RE_net_, SOR, and EC were approximately three times higher with SOB than without SOB. This indicates successful application of the SOB media to septic tanks and the process is very energy efficient. Moreover, H_2_S in water was rarely emitted into the atmosphere because of its oxidation upon SOB treatment. Therefore, SOB treatment could potentially replace aeration for H_2_S removal. The technique can be applied with efficiency and at low cost for wastewater treatment, even at large scales, with simultaneous prevention of environmental and health hazards that are caused due to H_2_S generation and spread.

## Figures and Tables

**Table 1:** Chemical properties of the examined solutions after experiments.

SOB Cultivation Period (Days)	pH	Residual SO_4_^2−^ in Solution (mg)	H_2_S Removed (mg)	SO_4_^2−^/H_2_S
Without SOB	7.2	32.1	111.3	0.29
5	7.1	33.8	113.2	0.30
12	6.9	35.6	115.1	0.31
23	6.3	88.3	168.3	0.52
30	6.0	176.2	275.4	0.64
45	5.7	232.6	327.3	0.71

**Table 2:** Aeration rate and DO concentration in previous studies and in this study.

Aeration Rate (L-Air/min/L-Water)	DO (mg/L)	Reactor	References
0.29	NS	Bioscrubber	[16]
0.28	0.5–1.0	Bioscrubber	[17]
0.59	>0.5	Bioscrubber	[18]
0.07	<0.2	Airlift bioreactor	[19]
0.37	NS	Packed bed bioreactor	[20]
0.13–0.38	-	-	this study

NS: Not specified.
